# INFANT MORTALITY AND FAMILY HEALTH STRATEGY IN THE 3^RD^ HEALTH REGIONAL OF PARANÁ, FROM 2005 TO 2016

**DOI:** 10.1590/1984-0462/2022/40/2020122

**Published:** 2021-05-14

**Authors:** Geovani Allan Broday, Ana Cláudia Garabeli Cavalli Kluthcovsky

**Affiliations:** a Universidade Estadual de Ponta Grossa, Ponta Grossa, PR, Brasil.

**Keywords:** Infant mortality, Family Health Strategy, Risk factors, Mortalidade infantil, Estratégia Saúde da Família, Fatores de risco

## Abstract

**Objective:**

To analyze the temporal trend in infant mortality and in populational coverage by the Family Health Strategy and associated factors with infant mortality in the municipalities of the 3^rd^ Health Regional of Paraná, Southern Brazil.

**Methods:**

Ecological time series study, with data from the Mortality Information System (*Sistema de Informação Sobre Mortalidade* - SIM), the Live Birth Information System (*Sistema de Informação Sobre Nascidos Vivos* - SINASC) and the Support Room for Strategic Management (*Sala de Apoio à Gestão Estratégica* - SAGE), from 2005 to 2016. Trends were calculated using polynomial regression. The associated factors with infant mortality were maternal, perinatal and obstetric variables. The significance level adopted was 5%.

**Results:**

Between 2005 and 2016, there were 115,796 births and 1,575 deaths of children under 1 year of age. Considering the municipalities together, the populational coverage by the Family Health Strategy went from 43.8% in 2005 to 66.4% in 2016 and the infant mortality from 17.1/1,000 live births in 2005 to 10.7/1,000 live births in 2016. The trend over time of populational coverage by the Family Health Strategy was crescent and of infant mortality was decrescent, for most municipalities. The factors associated with greater chances of death in children under 1 year of age were preterm gestational age (*Odds Ratio* - OR=15.05; 95% confidence interval - 95CI% 13.54-16.72), low birth weight (OR=15.14; 95%CI 13.61-16.84), multiple gestation (OR=4.51; 95%CI 3.74-5.45) and mother with up to 7 years of study (OR=1.93; 95%CI 1.74-2.14).

**Conclusions:**

Crescent trend in coverage by the Family Health Strategy was accompanied by a decrescent trend in infant mortality. The results can be a source of information for the strengthening of mother-child health actions, considering local and regional specificities.

## RESUMO

**Objetivo:**

Analisar a tendência temporal da mortalidade infantil e da cobertura populacional pela Estratégia Saúde da Família e os fatores associados à mortalidade infantil, nos municípios da 3ª Regional de Saúde do Paraná.

**Métodos:**

Estudo ecológico de série temporal, com dados do Sistema de Informação sobre Mortalidade (SIM), Sistema de Informações sobre Nascidos Vivos (SINASC) e Sala de Apoio à Gestão Estratégica (SAGE), de 2005 a 2016. As tendências foram calculadas por regressão polinomial. Os fatores associados à mortalidade infantil consistiram em variáveis maternas, obstétricas e perinatais. O nível de significância foi de 5%.

**Resultados:**

Entre 2005 e 2016, houve 115.796 nascimentos e 1.575 óbitos de menores de 1 ano. Considerando os municípios em conjunto, a cobertura populacional pela Estratégia Saúde da Família passou de 43,8% em 2005 para 66,4% em 2016, e a mortalidade infantil, de 17,1/1.000 nascidos vivos em 2005 para 10,7/1.000 nascidos vivos em 2016. A tendência ao longo do tempo da cobertura populacional pela Estratégia Saúde da Família foi crescente e a da mortalidade infantil decrescente para a maioria dos municípios. Os fatores associados a maiores chances de óbito em menores de 1 ano foram a idade gestacional pré-termo (*Odds Ratio* - OR=15,05; intervalo de confiança de 95% - IC95% 13,54-16,72), baixo peso ao nascer (OR=15,14; IC95% 13,61-16,84), gestação múltipla (OR=4,51; IC95% 3,74-5,45) e mãe com até sete anos de estudo (OR=1,93; IC95% 1,74-2,14).

**Conclusões:**

Tendência crescente da cobertura pela Estratégia Saúde da Família foi acompanhada de tendência decrescente da mortalidade infantil. Os resultados podem ser fonte de informações para o fortalecimento das ações em saúde materno-infantil, considerando as especificidades locais e regionais.

## INTRODUCTION

Infant mortality is considered a major public health concern in Brazil and involves biological, social, cultural, and health service failures.^1^ This indicator has been decreasing progressively in Brazil over the years,^2^ but, despite the reduction, regional differences remain in relation to infant mortality.^3^ ^,^ ^4^ ^,^ ^5^

Thus, the importance of reducing inequalities in mortality rates and achieving better levels of child survival is highlighted, through the accountability and commitment of health services to the population in its area of coverage.^1^ In this context, the Family Health Program was created, in 1994, which gradually became the main strategy for changing the care model, as well as expanding the initial access to services of the Unified Health System (*Sistema Único de Saúde* - SUS).^6^ Subsequently, it became the Family Health Strategy (FHS), because of its ability to reorganize the SUS care model, based on the principles of integrality and community participation in health services, as well as promoting health protection, promotion and recovery actions.^7^

The FHS has expanded considerably in the last 20 years.^6^ In 2001, there were only 5,421 Family Health teams in Brazil, compared to 42,784 in 2019, 2,277 of which were in Paraná.^8^ This expansion resulted, among other factors, in better access to health services and greater use of them, in addition to improvement in several indicators, ensuring greater equity and reducing health inequalities.^9^

Considering that the increase in coverage by the FHS is possibly associated with a reduction in the infant mortality rate,^3^ the analysis of infant mortality and the FHS is essential to better understand the health conditions of both maternal and infant populations. This allows for the development of priority actions and specific strategies for better organization of the health care network, with a focus on strengthening primary health care.

The aim of this study was to analyze the temporal trend of infant mortality and population coverage by the FHS and to verify the factors associated with infant mortality, in the municipalities of the 3^rd^ Health Regional of Paraná, from 2005 to 2016.

## METHOD

This is an ecological study of time series and techniques of spatial analysis of area. The state of Paraná is divided into 22 Health Regions, distributed into four macro-regions.^10^ The 3^rd^ Health Regional, located in the eastern macro-region, comprises 12 municipalities and covered an estimated population of 637,293 inhabitants in 2019, corresponding to 5.6% of the state’s population. Ponta Grossa has the largest population, with 351,736 inhabitants, while Porto Amazonas, the smallest one, with 4,848.^11^

In the Mortality Information System (*Sistema de Informação sobre Mortalidade* - SIM) and the Live Birth Information System (*Sistema de Informações sobre Nascidos Vivos* - SINASC)^12^, information was collected regarding the deaths of children under 1 year of age and live births of mothers residing, respectively, in the municipalities that make up the 3^rd^ Health Regional of Paraná, from 2005 to 2016. In the Support Room for Strategic Management (*Sala de Apoio à Gestão Estratégica* - SAGE),^8^ data were sought on the number of Family Health teams and the percentage of the population covered by the FHS, from 2005 to 2016. For comparison’s sake, data were collected from the entire state of Paraná.

Infant mortality coefficient was obtained by dividing the number of deaths of children under 1 year of age and the number of live births in the same location and period, expressed per 1,000 live births.

The calculation of the mean annual variation of population coverage by the FHS and infant mortality over the analyzed period was due to the difference between the value of the final year (2016) and that of the initial year (2005) divided by the number of years surveyed, *i.e.* 12 years.^3^

The trend of population coverage by the FHS for municipalities, for the 3^rd^ Health Regional and for Paraná, was made by the polynomial regression model,^13^ considering the percentages of coverage as the dependent variable and the years researched as the independent one. Thus, the second and third degree simple linear regression model were tested. The best model was chosen taking into account the analysis of the dispersion diagram, the value of the coefficient of determination (R^2^), and the analysis of the residuals. The variations in the series were smoothed by means of a moving mean centered on three successive means, except for the first and last years (two-year moving mean). The same procedures were performed to calculate the infant mortality trend.

The units of spatial analysis were the municipalities, considering the first and the last year surveyed, with infant mortality rates distributed in five groups, graduated in color scales. Spatial distribution was performed using the open source program TAB for Windows TabWin, developed by the Department of Informatics of the Unified Health System (*Departamento de Informática do Sistema Único de Saúde* - DATASUS).

The number of infant deaths in children under 1 year of age was compared to the number of live births in relation to maternal age (up to 19 years old or 20 years old or older) and mother’s education (up to seven years or eight years or more), obstetric variables on the type of pregnancy (multiple or single) and type of delivery (natural or cesarean) and perinatal referring to gender (male or female), gestational age 1 (preterm up to 36 weeks and 6 days of gestational age or term, from 37 to 41 weeks and 6 days), gestational age 2 (post-term 42 weeks or more of gestational age or term), birth weight 1 (low until 2,499g or normal from 2,500 to 3,999g) and birth weight 2 (normal or 4,000g or more). Ignored information were not considered. The association between variables was tested using Pearson’s χ^2^ test, calculating the Odds Ratio (OR) and the respective 95% confidence intervals (95% CI).

Data were processed using Microsoft Office Excel^®^ 2010 for Windows^®^, and the Statistical Package for the Social Sciences (IBM SPSS Statistics) software, version 15.0, was used for the calculations. The level of significance adopted was 5%. The project was submitted to the Research Ethics Committee and approved, under protocol number 3.067.224 and Certificate of Presentation of Ethical Appreciation (*Certificado de Apresentação de Apreciação Ética* - CAAE) number 03805718.4.0000.0105.

## RESULTS

In 2005, 6 of the 12 municipalities analyzed had over 50% of population coverage by the FHS, and in 2016 that number rose to nine municipalities. Considering the municipalities together, the population coverage by the FHS increased from 43.8% in 2005 to 66.4% in 2016 (Table 1).

**Table 1:** Percentage of population coverage by the Family Health Strategy, infant mortality and respective mean annual variations, between the initial (2005) and final years (2016).

Location	Population coverage by the Family Health Strategy (%)			Infant mortality**		
	Initial and final years		Mean annual variation	Initial and final years		Mean annual variation
	2005	2016		2005	2016	
Arapoti	100.0	87.7	-1.0	19.6	11.1	-0.7
Carambeí	19.9*	16.0	-0.3	22.4	5.5	-1.4
Castro	56.8	82.8	2.2	12.4	10.8	-0.1
Ipiranga	50.7	95.3	3.7	52.2	9.9	-3.5
Ivaí	57.1	76.0	1.6	15.3	10.8	-0.4
Jaguariaíva	30.4*	30.0	0.0	21.7	8.6	-1.1
Palmeira	43.7	92.0	4.0	27.5	9.1	-1.5
Piraí do Sul	14.6*	0.0	-1.2	19.4	13.7	-0.5
Ponta Grossa	34.4	79.7	3.8	14.6	10.9	-0.3
Porto Amazonas	100.0	72.2	-2.3	29.0	0.0	-2.4
São João do Triunfo	83.1	93.9	0.9	11.3	11.3	0.0
Sengés	18.6*	71.5	4.4	19.9	20.0	0.0
3^rd^ Health Regional	43.8	66.4	1.9	17.1	10.7	-0.5
State of Paraná	46.8	63.7	1.4	14.6	10.5	-0.3

*The implementation of Family Health started after 2005; **deaths in children under 1 year of age/1,000 live births.

All infant mortality coefficients were greater than 10 deaths per 1,000 live births (limit recommended by the World Health Organization) in 2005. In 2016, it was possible to observe a reduction in the coefficients in ten municipalities, five of them with rates below 10 deaths per 1,000 live births. Taking into account all municipalities together, infant mortality rose from 17.1/1,000 live births in 2005 to 10.7/1,000 live births in 2016 (Table 1).

There was a great variation in the infant mortality rates between the municipalities over the years, with the highest ones being 52.2 and 35.1 deaths per 1,000 live births and no deaths for some municipalities.

Most of the municipalities, totaling nine, showed an increasing trend in the coverage of the FHS, as well as the municipalities together (3^rd^ Health Regional) and Paraná. Only the municipalities of Porto Amazonas, São João do Triunfo, and Arapoti had a decreasing trend in coverage by the FHS (Table 2). Porto Amazonas exhibited high population coverage by the FHS (more than 70% coverage in all years surveyed), as well as São João do Triunfo and Arapoti (more than 70% coverage in most years).

**Table 2:** Trend in population coverage by the Family Health Strategy and infant mortality in the municipalities, in the 3^rd^ Health Regional and in the state of Paraná, from 2005 to 2016.

Location	Population coverage by the Family Health Strategy (%)				Infant mortality			
	Model*	R^2^	p-value	Trend	Model*	R^2^	p-value	Trend
Carambeí	Cubic	0.92	<0.001	Increasing	Cubic	0.90	<0.001	Decreasing
Ipiranga	Cubic	0.79	<0.01	Increasing	Cubic	0.81	<0.01	Decreasing
Ivaí	Cubic	0.81	<0.01	Increasing	Quadratic	0.79	0.001	Decreasing
Jaguariaíva	Quadratic	0.93	<0.001	Increasing	Quadratic	0.87	<0.001	Decreasing
Palmeira	Quadratic	0.93	<0.001	Increasing	Cubic	0.87	0.001	Decreasing
Ponta Grossa	Cubic	0.97	<0.001	Increasing	Cubic	0.89	<0.001	Decreasing
Sengés	Quadratic	0.94	<0.001	Increasing	Cubic	0.72	0.01	Decreasing
Castro	Quadratic	0.93	<0.001	Increasing	Cubic	0.35	0.30	Stable
Piraí do Sul	Cubic	0.75	<0.01	Increasing	Cubic	0.32	0.35	Stable
Porto Amazonas	Cubic	0.81	<0.01	Decreasing	Cubic	0.96	<0.001	Decreasing
São João do Triunfo	Quadratic	0.68	<0.01	Decreasing	Cubic	0.76	<0.01	Decreasing
Arapoti	Cubic	0.84	0.001	Decreasing	Cubic	0.32	0.34	Stable
3^rd^ Health Regional	Cubic	0.89	<0.001	Increasing	Quadratic	0.95	<0.001	Decreasing
Paraná	Cubic	0.99	<0.001	Increasing	Quadratic	0.99	<0.001	Decreasing

*Quadratic ($y=\beta_{0}+\beta_{1}x+\beta_{2}x^{2}$) and cubic ($y=\beta_{0}+\beta_{1}x+\beta_{2}x^{2}+\beta_{3}x^{3}$) polynomial regression models.

For infant mortality, nine municipalities showed a downward trend, except for Castro, Piraí do Sul, and Arapoti, with a stable trend. The municipalities together (3^rd^ Health Regional) and the state of Paraná also showed a decreasing trend (Table 2).

**Figure 1:**
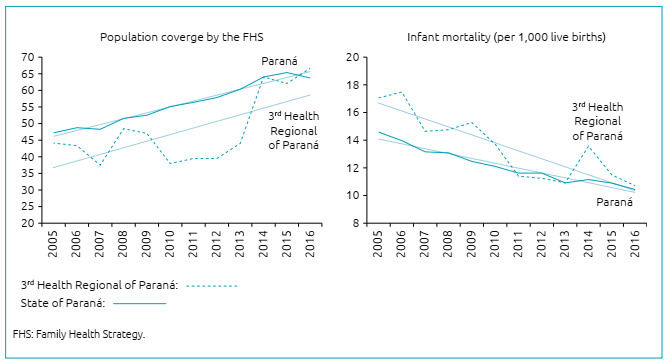
Curves and respective trend lines of population coverage by the Family Health Strategy (%) and infant mortality rates, per 1,000 live births, of the 3^rd^ Health Regional of Paraná and Paraná, from 2005 to 2016.

There was an increasing trend in population coverage by the FHS in the 3^rd^ Health Regional of Paraná, accompanied by a decreasing trend in infant mortality, as well as in the state of Paraná. Although the population coverage of the health regional was lower than that of the state of Paraná and the infant mortality rates were higher for the health regional in almost all the years studied, the values of these variables were very close between the health system and Paraná, in the last years analyzed (Figure 1).

**Figure 2:**
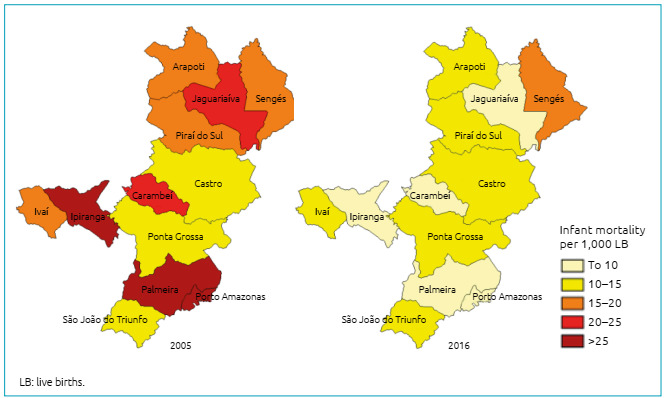
Infant mortality rates, per 1,000 live births, in the municipalities of the 3^rd^ Health Regional of Paraná, from 2005 to 2016.

Regarding the spatial distribution of infant mortality coefficients, it is observed that, in general, the coefficients were higher in the first year. In the last year there has been an improvement. For example, Carambeí, Jaguariaíva, Ipiranga, Palmeira and Porto Amazonas had less than 10 deaths for every 1,000 live births (Figure 2).

The variables that showed a significant association with infant deaths were: maternal age up to 19 years old, mother’s education up to seven years, multiple pregnancy, newborn male, preterm and post-term newborn, and low birth weight. The variables that showed, after adjustment, as a risk factor for infant deaths were: low birth weight (OR = 15.14), prematurity (OR = 15.05), multiple pregnancy (OR = 4.51), and mother with up to seven years of study (OR = 1.93) (Table 3).

**Table 3:** Distribution of absolute and relative frequencies of infant deaths and total live births in the municipalities of the 3^rd^ Health Regional of Paraná, according to maternal, obstetric and perinatal variables, from 2005 to 2016*.

	Infant deaths n (%)	Live births n (%)	p-value	Odds Ratio (95%CI)
Age of the mother (years)**				
Up to 19	395 (26.6)	24,835 (21.4)	<0.001	1.33 (1.18-1.49)
20 or more	1,088 (73.4)	90,961 (78.6)		
Education of the mother (years)**				
Up to 7	663 (45.5)	34,814 (30.2)	<0.001	1.93 (1.74-2.14)
8 or more	793 (54.5)	80,445 (69.8)		
Type of pregnancy **				
Multiple	124 (8.2)	2,249 (1.9)	<0.001	4.51 (3.74-5.45)
Single	1,386 (91.8)	113,467 (98.1)		
Type of delivery**				
Vaginal	770 (51.8)	57,269 (49.5)	0.07	1.10 (0.99-1.22)
Cesarean	716 (48.2)	58,452 (50.5)		
Gender**				
Male	851 (54.1)	59,279 (51.2)	0.02	1.12 (1.02-1.24)
Female	723 (45.9)	56,512 (48.8)		
Gestational age 1**				
Preterm	824 (56.2)	8,943 (7.9)	<0.001	15.05 (13.54-16.72)
Term	642 (43.8)	104,859 (92.1)		
Gestational age 2**				
Post-term	16 (2.4)	1,570 (1.5)	0.04	1.66 (1.01-2.74)
Term	642 (97.6)	104,859 (98.5)		
Weight at birth 1**				
Low	893 (60.6)	10,221 (9.2)	<0.001	15.14 (13.61-16.84)
Normal	581 (39.4)	100,662 (90.8)		
Weight at birth 2**				
Normal	581 (95.9)	100,662 (95.3)	0.53	1.13 (0.76-1.69)
High	25 (4.1)	4,911 (4.7)		

*χ^2^ test was used; ** the following information was ignored: ignored: mother’s age (n=0 in live births/92 in deaths), mother’s education (n=537 in live births/119 in deaths), type of pregnancy (n=80 in live births/65 in deaths), type of delivery (n=75 in live births/89 in deaths), gender (n=5 in live births/1 in deaths), color/race of birth (n=579 in born alive/56 in deaths), gestational age (n=424 in live births/93 in deaths), and birth weight (n=2 in live births/76 in deaths); 95% CI: 95% confidence interval.

## DISCUSSION

Considering the municipalities of the 3^rd^ Health Regional of Paraná together, as well as the state of Paraná, there was an increasing trend in population coverage by the FHS, which was accompanied by a decreasing trend in infant mortality. Among the municipalities analyzed, the majority of them (seven) also showed an increasing trend in population coverage by the FHS and a decreasing one in infant mortality. The other two municipalities with an increasing trend in coverage showed a stable trend in infant mortality.

Studies have reported increased FHS coverage over the years.^3^ ^,^ ^5^ ^,^ ^9^ Municipalities with high FHS coverage have greater use of primary health services, in addition to faster acceleration in health indicators such as the mortality of infants and children under 5 years of age, reduction in hospitalizations for preventable causes by primary care and mortality from cardiovascular and cerebrovascular causes.^14^

Literature has shown a decreasing trend in infant mortality in all macro-regions of the state of Paraná between 2000 and 2014^4^ and in the state of São Paulo.^15^ This decline has also been observed worldwide, although many regional disparities still persist.^2^ Some developed countries have infant mortality rates close to two per 1,000 live births, while many countries in sub-Saharan Africa reach values above 60 per 1,000 live births,^16^ highlighting inequities in access to health around the world, reflecting social and economic inequalities.

The association between greater coverage by the FHS and improvement in health indicators, including infant mortality, has been described in the literature.^3^ ^,^ ^5^ ^,^ ^14^ In the present study, the increase in FHS coverage was accompanied by a reduction in infant mortality for municipalities together and for most municipalities, except in two, with stable infant mortality. Three municipalities showed a decreasing trend in coverage, two with a decreasing trend and one stable for infant mortality. It is important to note that these three municipalities maintained a high population coverage by the FHS (greater than 70%) during most years, which may partly justify the fact that infant mortality has remained decreasing or stable.

In the Brazilian semiarid, the *Bolsa Família Program*, allied to the FHS, significantly reduced infant mortality and total fertility rates.^17^ In the state of São Paulo, between 1998 and 2009, FHS coverage above 50% showed a protective effect in relation to post-neonatal mortality, and coverage of up to 50% or higher were protective factors for hospitalizations for pneumonia in children under 1 year. The authors also concluded that the effectiveness of the FHS on child health outcomes may vary, due to local and regional contexts.^5^

In an analysis between infant mortality and population coverage by the FHS in the Federation units, from 1998 to 2008, it was observed that the expansion of coverage by the FHS was associated with the reduction of infant mortality rates in 73% of Brazilian states, with differences in rates between states and regions.^3^

Based on a longitudinal study evaluating the relationship between doctors in primary care and infant mortality in Brazil, between 2005 and 2012, it was estimated that the increase of one doctor in primary care for a population of 10,000 people was associated with less 7.08 deaths of children under 1 year old per 10,000 live births, showing the importance of primary care as a key component for the creation of a quality and universal health system.^18^

In fact, the expansion and adequacy of the FHS allowed better access to health services and greater use of them, reduced child and adult mortality and expanded access to treatments, infrastructure and knowledge, in addition to reducing unnecessary hospitalizations, ensuring greater equity and reducing health inequalities.^9^

When characterizing the deaths of children under 1 year of age in the 3^rd^ Health Regional of Paraná, the variables that were most likely to occur were identified, namely, low birth weight, preterm newborn, multiple pregnancy, and maternal education of up to seven years.

In this study, low birth weight and prematurity were factors strongly associated with infant mortality. Newborns with low birth weight had 15.14 times more chance of death with less than 1 of life, and preterms, 15.05 times more chance. In a case-control study on risk factors for infant mortality in five Brazilian cities, low birth weight had a strong association with deaths in children under 1 year old in all analyzes performed, remaining in the final model of the five cities.^19^

Considering that prematurity is an important factor for infant mortality, the causes for premature birth must be identified, in order to be avoided.^20^ In Florianópolis, the chance of neonatal death was 6.09 times higher in premature newborns and 9.46 times higher in those with low birth weight.^21^ Other studies have also reported an association between infant mortality and prematurity and low birth weight,^22^ ^,^ ^23^ emphasizing the importance of care for high-risk pregnant women, seeking to reduce the incidence of both factors.

The occurrence of multiple pregnancies presented 4.51 times more chance of death with less than 1 year of life, in the municipalities of the 3^rd^ Health Regional of Paraná. Na association between infant mortality and multiple pregnancy has also been demonstrated in other studies.^22^ ^,^ ^24^ In the United States, the risk of mortality increased according to the number of fetuses in pregnancy: infant mortality for twins was approximately four times higher; for triplets, 12 times; and for quadruplets, 26 times compared to the birth of a single child.^25^ In addition, premature live births and low birth weight are more frequent in multiple pregnancies, reinforcing the importance of special attention for this higher-risk patient profile.^22^

Low education was identified as a risk factor for infant mortality in a study carried out in the 9^th^ Regional Health Department of Paraná, between 1997 and 2008, and may reflect the mother’s low socioeconomic level, which leads to greater maternal and child risk, as it makes access to information and guidance more difficult, complicating care and assistance.^23^ In the Southern Region, between 2011 and 2012, children of mothers with less than eight years of study had an 85% higher chance of death before completing 1 year of life, compared to children of mothers with more than eight years of study.^26^

Post-term birth was also more frequently associated with infant death. Although less studied, prolonged pregnancy should be remembered, as it is the cause of preventable death, and can be reduced with appropriate care.^1^

Teenage pregnancy (up to 19 years of age) was associated with infant mortality, in a previously mentioned study^23^ and in an infant mortality assessment in Londrina, Paraná, in 2000/2001 and 2007/2008.^24^ Fertility among adolescents is influenced by several factors, such as greater income inequalities, among others.^27^ It is important to highlight the need to offer preventive health actions to adolescents, especially family planning, to avoid unwanted pregnancies in this age group, as well as offering adequate assistance to pregnant adolescents.

Developing countries with high socioeconomic inequality, such as Brazil, should monitor trends in neonatal and under-5 mortality and accurately target health and intersectoral policies, as it is known that poor municipalities have worse health care than the richest.^28^ In this context, measures have been implemented to reduce child mortality, such as the *Rede Mãe Paranaense* Program, which since 2012 proposes health promotion actions during pregnancy and the puerperium, with monitoring of the child’s development, especially in the first year of life.^29^ It is necessary to recognize situations of risk, appropriate and resolutive care to pregnant women in prenatal, at childbirth, and to children, at birth, in surveillance, in health promotion, and adequate assistance. These are basic actions with great potential to improve children’s survival and quality of life.^1^

One of the limitations of the present study was the fact that data on infant mortality may have been influenced by other factors or programs that were not considered. In addition, as the study was carried out in only one health region, the results cannot be generalized to the other regions of Paraná, due to the specificities of each one. There was also the use of secondary data, with the possibility of underreporting deaths, however this limitation can be mitigated considering that SINASC data have high coverage, completeness, and reliability.^30^

This study contributed to a better understanding of the infant mortality profile, population coverage by the FHS and associated factors in the municipalities of the 3^rd^ Health Regional of Paraná. The results presented can be a source of information for directing specific strategies aimed at strengthening health actions, at all levels of care, especially in the FHS, to improve maternal and child health indicators, with a more accessible health system, with less inequities and greater resolution.
