# ERRATUM

**DOI:** 10.1590/1984-0462/2022/40/2020122erratum

**Published:** 2023-07-17

In the manuscript “Infant mortality and Family Health Strategy in the 3rd health regional of Paraná, from 2005 to 2016”, DOI: 10.1590/1984-0462/2022/40/2020122, published in the Rev Paul Pediatr. 2022;40:e2020122.

**On page four, second column, third paragraph:**

Where it reads:

The variables that showed, after adjustment, as a risk factor for infant deaths were: low birth weight (OR = 15.14), prematurity (OR = 15.05), multiple pregnancy (OR = 4.51), and mother with up to seven years of study (OR = 1.93) (Table 3).

It should read:

The variables associated with greater chances for infant deaths were: low birth weight (OR = 15.14), prematurity (OR = 15.05), multiple pregnancy (OR = 4.51), and mother with up to seven years of study (OR = 1.93) (Table 3).

